# Reconstruction of regulatory networks through temporal enrichment profiling and its application to H1N1 influenza viral infection

**DOI:** 10.1186/1471-2105-14-S6-S1

**Published:** 2013-04-17

**Authors:** Elena Zaslavsky, German Nudelman, Susanna Marquez, Uri Hershberg, Boris M Hartmann, Juilee Thakar, Stuart C Sealfon, Steven H Kleinstein

**Affiliations:** 1 Center for Translational Systems Biology and Department of Neurology, Mount Sinai School of Medicine, New York, NY 10029, USA; 2 Department of Pathology, Yale University School of Medicine, New Haven, CT 06510, USA; 3 Interdepartmental Program in Computational Biology and Bioinformatics, Yale University, New Haven, CT 06510, USA; 4 Department of Biology, York University, Toronto, Ontario, Canada M3J 1P3; 5 School of Biomedical Engineering, Science and Health Systems, Drexel University, Philadelphia, PA 19104, USA

## Abstract

**Background:**

H1N1 influenza viruses were responsible for the 1918 pandemic that caused millions of deaths worldwide and the 2009 pandemic that caused approximately twenty thousand deaths. The cellular response to such virus infections involves extensive genetic reprogramming resulting in an antiviral state that is critical to infection control. Identifying the underlying transcriptional network driving these changes, and how this program is altered by virally-encoded immune antagonists, is a fundamental challenge in systems immunology.

**Results:**

Genome-wide gene expression patterns were measured in human monocyte-derived dendritic cells (DCs) infected *in vitro *with seasonal H1N1 influenza A/New Caledonia/20/1999. To provide a mechanistic explanation for the timing of gene expression changes over the first 12 hours post-infection, we developed a statistically rigorous enrichment approach integrating genome-wide expression kinetics and time-dependent promoter analysis. Our approach, *TI*me-*D*ependent *A*ctivity *L*inker (TIDAL), generates a regulatory network that connects transcription factors associated with each temporal phase of the response into a coherent linked cascade. TIDAL infers 12 transcription factors and 32 regulatory connections that drive the antiviral response to influenza. To demonstrate the generality of this approach, TIDAL was also used to generate a network for the DC response to measles infection. The software implementation of TIDAL is freely available at http://tsb.mssm.edu/primeportal/?q=tidal_prog.

**Conclusions:**

We apply TIDAL to reconstruct the transcriptional programs activated in monocyte-derived human dendritic cells in response to influenza and measles infections. The application of this time-centric network reconstruction method in each case produces a single transcriptional cascade that recapitulates the known biology of the response with high precision and recall, in addition to identifying potentially novel antiviral factors. The ability to reconstruct antiviral networks with TIDAL enables comparative analysis of antiviral responses, such as the differences between pandemic and seasonal influenza infections.

## Background

Pathogenic viruses, such as influenza and measles, subvert normal immune functioning through the expression of immune antagonists, such as the influenza NS1 protein. These antagonists differ between viral strains, and are crucial components of viral pathogenicity. Determining how these antagonists interact with the host immune system would be aided by knowledge of the genetic regulatory network that operates in response to infection. Recently, multiple studies have begun to define the cellular response to bacterial and viral pathogens in specific cell types (see [1-6] and references therein). However, despite the important role that regulatory networks play in orchestrating immune responses, their constituent transcription factors (TFs) and architectures remain largely unknown for most systems.

Computational methods [7-17] that analyze high-throughput experimental data have proven very useful in helping define transcription regulatory networks [18], but, with most of these studies done in yeast or bacteria, applications to mammalian systems are still lacking. Moreover, previous approaches are often based on the assumption that genes sharing a similar temporal expression profile are regulated by common transcription factors. Such methods cluster genes based on similarity of expression profiles over the entire time-course. While plausible, the criterion of expression similarity across the full time-series can be unnecessarily restrictive. Especially in mammals, where mRNA levels following initial up-regulation can be greatly affected by a variety of post-transcriptional regulatory mechanisms (e.g. miRNAs [19]), there is less reason to expect good conservation across the entire duration of the time-series.

Here, we develop an integrative, time-centric method called TIDAL (*TI*me-*D*ependent *A*ctivity *L*inker) that focuses on uncovering dynamic transcription regulatory programs, and apply this approach to antiviral responses. In contrast to other approaches, we consider initial up-regulation time as the main criterion to identify genes with common regulatory control logic. TIDAL is an integrative method, relying on expression data and promoter binding site information conserved across species for inference of regulatory relationships. Note that since each individual data type can be incomplete or error-prone, integrative methods provide more robust and accurate results by drawing on multiple lines of evidence and requiring consistency between several heterogeneous source of data [20]. Furthermore, the association of TF activity with specific time windows together with the expression dynamics of the particular TF gene allows TIDAL to produce a temporally-driven map that derives time-dependent profiles for each factor's regulatory activity. The integration of time into the analysis is a critical component that has aided our understanding of transcriptional networks [3,13,21-23] by better determining when transcription factors exert their influence in propagating regulatory signals. Finally, TIDAL's ability to create a global, maximally inclusive view of the transcriptional network is an advantage over existing methods that isolate individual regulatory modules without connecting them into a unified temporally-aware transcriptional cascade. The software implementation of TIDAL is freely available at http://tsb.mssm.edu/primeportal/?q=tidal_prog.

We use TIDAL to learn the transcriptional network underlying the response of human DCs to infection with seasonal H1N1 influenza A/New Caledonia/20/1999. While some components of the regulatory network controlling the response to influenza are well-known, like the critical roles played by IRF and STAT family transcription factors [24], our understanding of the larger regulatory map remains limited. When applied to an infection time course, TIDAL produces a global temporal view of the transcription regulatory network underlying the antiviral response. The network successfully recapitulates known biology, identifying the IRF, STAT and NFkB factors as important regulators, and accounting for observed expression changes in the majority of up-regulated genes. We also apply TIDAL to study the response to infection with measles [25], another important human virus, with similarly encouraging results. Along with the regulatory linkages connecting the known antiviral transcription factors, key roles are predicted for several regulators with no known role in antiviral responses. The ability to uncover regulatory networks from infection time-series data will greatly improve our understanding of the myriad ways that pathogenic viruses subvert normal immune function, providing insight into their pathophysiology and potentially aiding in the development of new therapeutic strategies.

## Results and discussion

The input to TIDAL consists of a gene-expression time-series. Internally, TIDAL also makes use of TRANSFAC transcription factor binding site descriptions and genomic multiple alignments with transcription start site annotations for TF binding site filtering. The method constructs a regulatory network using the following basic steps, with greater implementation details provided in the *Methods *section:

1. Identify up-regulated genes in the gene expression time-series, and group genes according to the time of their first detected up-regulation (*t*).

2. Infer transcriptional regulators that are active at each *t *by identifying TRANSFAC matrices with predicted targets that are over-represented in the group of genes first up-regulated at time *t*.

3. Define an activity window for each TF as the union of time-points when: (i) the TF gene is up-regulated and (ii) any TRANSFAC matrix annotated to the TF has inferred activity.

4. Connect TFs to targets that contain a TF binding site and are first up-regulated within the TF's activity window. For each target, retain the top few (can be set as a parameter) incoming links ranked by their enrichment P-values.

In the following sections, we use TIDAL to infer the transcriptional regulatory networks that control the immune responses to influenza and measles infections in human monocyte-derived dendritic cells. In each case, infection of these cells triggers a genetic regulatory cascade that controls the differential expression of hundreds of genes. The qualitative results obtained from the application of TIDAL to both datasets are similar. We present our analysis of the influenza infection in the main body of the paper, while analogous results for the measles infection are contained in the Supplementary Information.

### Identification of transcription factors driving the influenza response

**Figure 1:**
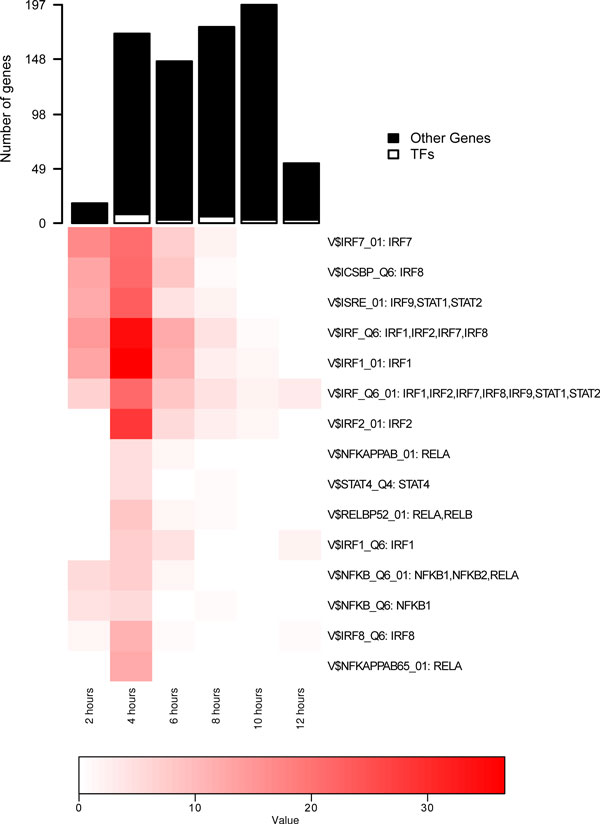
**Enrichment profiles for TFs implicated in the influenza response**. Top panel shows numbers of genes first up-regulated at each time-point, split by TF and non-TF genes. Bottom panel shows a heatmap plot with the over-representation analysis of targets associated with TRANSFAC matrices (rows) over time (columns). Only matrices with inferred activity are pictured, and the color (-*log*(P-value)) indicates the significance of the hypergeometric test. The associations between a TRANSFAC matrix and the individual (up-regulated) TFs are shown to the right of the color profile.

Human monocyte-derived DCs were infected in vitro with seasonal H1N1 influenza A/New Caledonia/20/1999. Genome-wide mRNA expression analysis was carried out at 0, 2, 4, 6, 8, 10 and 12 hours post-infection. We identify a set of 763 genes that are up-regulated over the course of the response (Figure 1, top).

The first step in reconstructing the antiviral response network is to infer the set of TFs that likely regulate these observed gene expression changes. TFs act through distinct cis-regulatory elements located in the promoter regions of their target genes. We assume that genes which are up-regulated at similar times share cis-regulatory logic (i.e., they are regulated by common TFs). This is an important distinction from many other methods (e.g. [9,13]), which assume common transcriptional regulation for groups of genes sharing similarity in expression across their entire time-series profiles. We propose that this is likely to be unnecessarily restrictive, and instead infer the identity of the regulators of each distinct time-point by looking for TFs whose target genes are over-represented among the genes up-regulated at that time. Note that we do not attempt to explain the process of down-regulation. We have previously observed that the timing of expression changes among down-regulated genes is not correlated across experimental replicates, suggesting looser regulatory control [26]. Our goal is thus to explain the more tightly controlled up-regulation events using the set of transcription factors whose genes themselves are transcriptionally regulated as part of the response. These factors are key candidates for propagation of transcriptional signals [3].

While genome-wide identification of direct TF targets can be done experimentally using techniques such as ChIP-Seq [27], it has only been done for few TFs and limited to specific experimental conditions. Instead, we identify TF targets computationally, and use the presence of binding sites as a proxy for each potential regulatory relationship between a TF and target. We begin with the set of 744 TF binding signatures annotated to human proteins within the TRANSFAC database, a broad compilation of experimentally verified binding sites summarized as position weight matrices [28]. We consider any gene that contains a binding site described by that factor's TRANSFAC [28] matrix in its promoter region a target. We also require that the binding site be evolutionarily conserved, since conservation has been shown to reduce rates of false positive binding site prediction [29].

Genes are grouped according to the time of their first detected up-regulation in the microarray (Figure 1, top panel). There is one group for each of the microarray sampling times: 2, 4, 6, 8, 10 and 12 hours post-infection for the influenza dataset analyzed here. For each of these groups, over-representation analysis, comparing the number of genes in the group with a specified TF binding site against a background set via the hypergeometric distribution (see *Methods*), is applied to infer the activity of transcription factors. Of the 49 TRANSFAC matrices annotated to genes that are up-regulated at some point in the time-series, our analysis identifies 15 of these matrices as having a role in the response. Figure 1 provides a visual display of the inferred activity for each of these TRANSFAC matrices over time. The set of transcription factors associated with these matrices contains many known integral components of the antiviral response, including those linked with interferon activation (IRFs, STATs, NFkB) [24]. Indeed, these factors feature prominently in the activation heatmap, with the IRFs and some STAT factors showing sustained activity over a long period. The inferred activity for most TFs occurs early in the response, indicating a rapid transition to the antiviral state following infection. Figure 1 also shows the highly redundant nature of the TRANSFAC database, with many matrices annotated to multiple, overlapping sets of TF genes.

**Figure 2:**
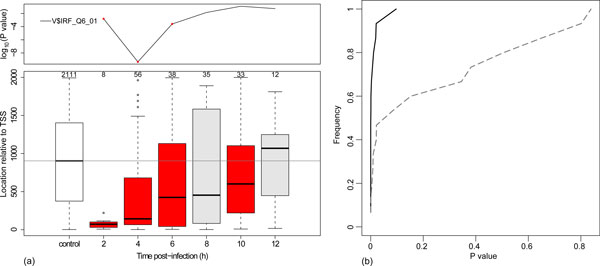
**Binding site location mirrors TF activity profile**. (a) Analysis of the general IRF matrix (V$IFR_Q6_01). Top panel shows profile for the over-respresentation analysis, with red dots indicating significance at 2, 4, and 6 hours post infection (FDR *q <*0.05). Bottom panel shows Boxplots of the absolute locations of binding sites in promoter regions of genes first up-regulated at each time-point. The number of genes in each group is indicated above the box. The medians are indicated by horizontal bars. The box at time zero shows a control group of genes, and contains all RefSeq genes with binding sites for the same matrix that are not differentially-expressed. Red bars indicate that the median binding site position is significantly closer to the TSS as compared with the control group (*P <*0.05, Mann-Whitney test). When multiple binding sites for a matrix are present for a single gene, the one closest to the TSS is selected. (b) Plots the cumulative distributions of the P-values, testing for differences in binding site locations as described in (a), at the time of minimum enrichment P-value (highest inferred TF activity, solid line) and maximum enrichment P-value (least inferred TF activity, dashed line). We observe a significant shift in the locations distribution (*P <*0.0002, Mann-Whitney test) toward the TSS during times of TF activity.

We validate the inferred activity profiles through a complementary computational analysis. It has been observed that the location of functional cis-regulatory binding sites relative to the transcription start site (TSS) is non-random [30,31]. True cis-regulatory binding sites are often located close to the TSS. Thus, we predict that the location of the binding sites for active TFs would be correlated with their inferred activity profiles, with binding sites located closer to the TSS during times when the activity of the associated TF is inferred. As an example, binding sites for the V$IRF_Q6_01 matrix are located significantly closer to the TSS in genes that are up-regulated at 2, 4, 6 and 10 hours post-infection (*P <*0.05, Mann-Whitney test) (Figure 2a, bottom left panel). This pattern closely parallels the temporal activity profile for the IRF matrix, which is significant at 2, 4 and 6 hours post-infection Figure 2a, top left panel). Furthermore, the median distance of the site from the TSS steadily increases over time so that by 12 hours post-infection, the locations of the IRF binding sites are not different from the control set, suggesting that IRF-driven activity is minimal at this point. To test whether this pattern holds for the other TRANSFAC matrices with inferred activity, we compare the shift in binding site locations at the time of peak predicted activity (e.g., 4 hours post-infection for the IRF matrix) with the shift at the time of minimum activity. As seen in Figure 2b, we observe that binding sites show a significantly shifted location distribution when the associated TF is maximally active (*P <*0.0002, Mann-Whitney test). Inspection of the location plots shows that the shift in location during times of peak TF activity is towards the TSS, as expected.

### The influenza transcriptional response network

Having identified the set of TFs driving the antiviral response to influenza, we next seek to explain how each of the individual TFs becomes up-regulated by connecting these factors into a coherent network. We initially consider all TF pairs such that a binding site of one factor is located in the promoter region of the other based on our promoter analysis. We filter these potential network links in two steps. First, we define a time-window for each TF's activity based on when its mRNA is up-regulated, and when any of its associated TRANSFAC matrices shows significant activity, and retain only regulator-target connections where the target is up-regulated within the regulator's inferred activity window. Since TF binding site locations are correlated with the TF activity profiles (Figure 2), limiting links to the activity windows allow us to have greater confidence in the inferred regulatory relationships. Second, to predict the most likely regulators for each target, we rank each target's incoming links by the regulator's closest time of enrichment, and retain the top three links in the network, breaking ties by enrichment P-value (see *Methods *for details).

**Figure 3:**
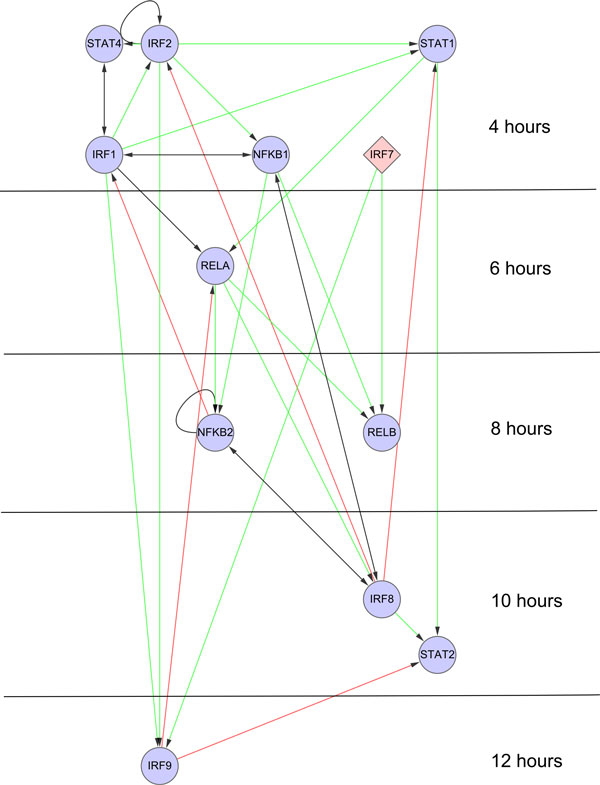
**The influenza regulatory response network**. Network nodes correspond to individual TFs. Edges indicate predicted regulatory relationships, which can be either forward (green links), feed-back (red links) or reciprocal (black links). Time in the figure progresses vertically down, with nodes placed in the time-slice during which the gene is first differentially expressed. Diamond-shaped nodes indicate TFs with no predicted regulators. The network edges are filtered to include at most three predicted regulators for each target.

To visualize the inferred network, we order nodes (representing individual TFs) vertically based on their up-regulation times. Links between nodes indicate predicted regulatory relationships (Figure 3). Since each TF can be associated with multiple TRANSFAC matrices, there are several choices of how to place a TF in the network. We choose to place each TF based on the time its mRNA was first up-regulated in the microarray time-series. Alternate placement of TFs, based on their associated TRANSFAC matrix enrichment, is ruled out for two main reasons. First, many of the matrices are enriched at multiple time-points, making the choice of node placement somewhat arbitrary. Second, this scheme does not allow for differentiation between TFs that are annotated to the same TRANSFAC matrix, making disambiguation of which factor is actually driving the response difficult.

Having filtered the links by TF activity windows and having limited each node to three most likely regulators, we obtain an influenza antiviral network that contains 12 TF nodes and 32 regulatory links (Figure 3). We visualize it with Cytoscape [32] using the Cerebral [33] plug-in to produce a time-dependent layout. To interpret the network visualization in Figure 3, it is helpful to keep in mind that links connect regulators to targets, and that arrow-tails indicate up-regulation of the regulator itself, while arrow-heads indicate transcriptional activity of the regulator. A majority of the links in the network are forward links (colored green), which propagate the transcriptional signal forward through time. Other links (colored red), indicate regulatory feedback relationships, which may contribute to prolonged activity of a TF that was first activated earlier. Within each time slice the TF nodes are laid out in such a way as to enable the forward links to point downward. Surprisingly, all the predicted TFs could be connected together into a single network, even after a large portion of the edges have been removed by the filtering procedure. Furthermore, considering all of the putative targets of TFs in the network, we account for 53% of the genes that are up-regulated during the influenza response.

### Performance comparison

A large number of methods have been proposed for inference of gene regulatory interactions based on time-series gene expression data [34]. Many of these methods rely heavily on knock-out (or knock-down data) [35-37], or require large microarray compendia [11], and are thus not appropriate for the experimental setup considered here. To evaluate the performance of TIDAL, we sought another method that could predict regulator-target pairs and associate these with specific time-points, features that we consider essential benefits of TIDAL. While several approaches were considered [7,8,16], we have chosen to compare TIDAL's results with those of the Dynamic Regulatory Events Miner (DREM) [13], another state-of-the-art computational approach that can operate on a single time-series dataset. DREM is a method that infers global regulatory networks, assigning transcription factors to individual time-points, and thus allows for a direct comparison of the inferred regulators along with their predicted timing between the two methods. It is important to note that while both DREM and TIDAL rely on inferring response regulators by testing for statistical enrichment of putative targets among differentially regulated genes, a major difference lies in the grouping of genes being tested. TIDAL groups genes by time of first differential expression, and DREM performs a clustering based on the full temporal profile, identifying genes with similar expression patterns across the entire time-series.

**Figure 4:**
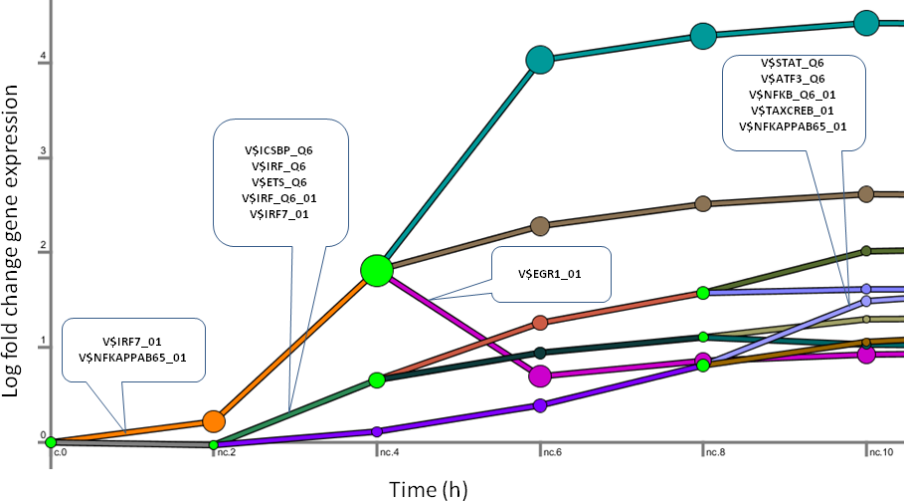
**Dynamic regulatory map of the influenza response**. DREM [13] is used to create a dynamic map based on the time-series fold-change gene expression data as described in *Methods*. DREM parameters are left at their defaults. Each line in the figure represents a temporal cluster. Predicted regulatory events (cut-off of 10^−3^) are indicated pointing to the temporal profile immediately after the split they regulate.

DREM identifies points in the time-series where the expression of a subset of genes diverges from the rest. It is assumed that this divergence is the result of transcriptional control mechanisms and DREM associates the divergence events with TFs that regulate them in order to produce a global temporal map. It is also important to note that DREM can identify regulation events that are beyond the scope of our method, such as declines in expression following the initial up-regulation event. Applied to the influenza gene expression data and using the same set of TF matrices and their mapped binding sites as in TIDAL, DREM identifies 9 distinct temporal profiles (see Figure 4), with 13 TRANSFAC matrices regulating 4 of the 9 clusters leading out of different branching points. To compare the relative performance of the methods, we compute the overlap between the TFs inferred by TIDAL (pictured in Figure 3) and DREM (mapped from the TRANFAC matrices pictured in Figure 4) with a set of TFs with 'known' immune involvement. This set of known genes consists of TFs that are part of the general pathogen response signature [1] and the core dendritic cell response [38]. High overlap with this set serves as a good indicator of correctness for a method's implication of genes as involved in an influenza or another infection.

**Figure 5:**
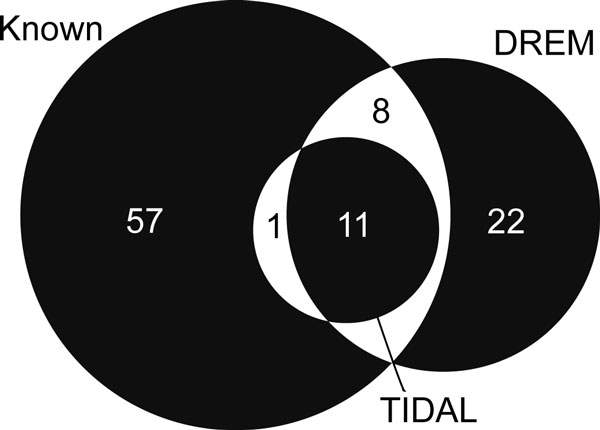
**Transcription factor overlap comparison with DREM for the influenza response**. The inferred TFs from TIDAL (Figure 3) and DREM (Figure 4) are compared to a set of TFs with implicated immune response roles. This 'known' set consists of the union between two external gene signatures: the general pathogen response signature defined in [1] and the core dendritic cell response signature defined in [38], of which only TF are included. The TF list is derived using a mapping of gene symbols in [43] along with the set of genes linked to human TRANSFAC matrices (see *Methods*).

While many more transcription factors, when mapped from TRANSFAC matrices, are predicted by DREM as compared to TIDAL, taken together, they count among their targets approximately 69% of all up-regulated genes (compared with 53% of genes explained by TIDAL). Recall for the two methods (see Figure 5) stands at 16% for TIDAL and 25% for DREM. However, since we do not expect that all of the 'known' TFs are involved in the influenza response in dendritic cells, these recall values are likely to be underestimated for both methods. In contrast, TIDAL has much higher precision (100%) compared with DREM (40%). It is important to note that these results for the DREM analysis depend on the preprocessing of the microarray data. When DREM is supplied with the full set of genes without differential expression filtering, it produces much inferior results. Moreover, filtering the inferred transcription factors for differential expression, a step we consider essential to our analysis, also improves DREM's precision (to over 90%) without significantly reducing the recall rate.

DREM identifies the most well-known TF families in the antiviral response (namely IRF, STAT and NFkB). However, NFkB and STAT activity is predicted to regulate the latter stages of the response (via the V$STAT_Q6 and V$NFKB_Q6_01 matrices). In contrast, TIDAL predicts their activity early in the response, which is consistent with known biology [24]. Overall, TIDAL's ability to more precisely infer the important antiviral regulators among the pathogen response signature genes coupled with more accurate temporal identification of their regulatory activity point to its better performance.

## Conclusions

In this study, we describe the Time-Dependent Activity Linker (TIDAL), a bioinformatics method for antiviral network inference that integrates a statistically rigorous enrichment approach with genome-wide expression kinetics and time-dependent promoter analysis. We use TIDAL, in combination with new experiments, to define the regulatory networks that operate in DCs infected with seasonal H1N1 influenza virus and Measles virus. DCs provide a crucial link between virus detection and adaptive immunity, and the ultimate success of antiviral responses depends on the early signaling and maturation elicited in these cells. The DC response to virus infection depends on the activation of multiple pathways, which is carried out by a complex genetic regulatory program. This program can be altered by viral immune antagonists, such as the NS1 protein of influenza [39,40]. In conjunction with time-series gene expression measurements, TIDAL can be used to study the function of these antagonists by comparing the networks elicited by different viral strains.

**Figure 6:**
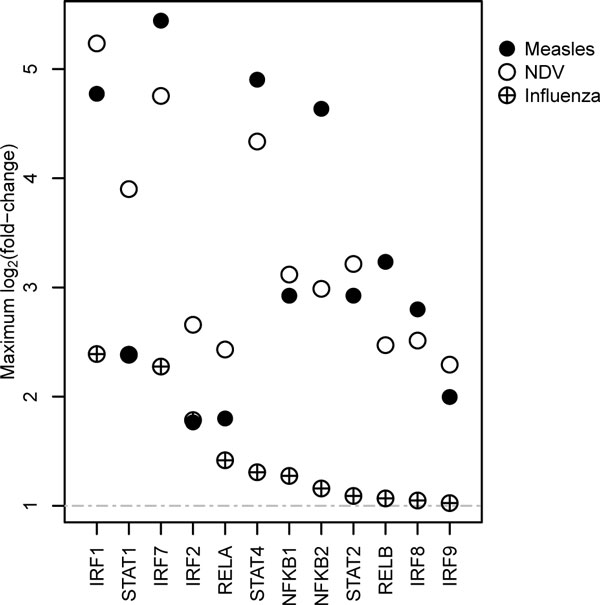
**Comparison of the influenza response with measles and NDV responses in human dendritic cells**. The expression fold-changes for TFs in the inferred influenza network are compared to other responses: NDV [26] and measles [25]. The TFs are sorted by decreasing fold-change in the influenza response. For each gene, the maximum fold-change over all measured time-points with detected expression is plotted.

The networks produced by TIDAL consist of a set of transcription factors, their temporal activity profiles, and specific regulatory relationships. We apply TIDAL to reconstruct the transcriptional network mediating the antiviral program in human DCs in response to influenza and measles infections. TIDAL reliably captures known elements common to many antiviral responses, including the key roles of the NFkB, IRF and STAT family transcription factors. When compared with DREM, another state-of-the-art method for identifying response regulators, TIDAL has similar recall, but higher precision. In fact, all of the TFs identified by TIDAL in the influenza network have previously been connected to the immune response, and all of the TFs are also up-regulated in DCs during other anti-viral responses (Figure 6). This consistency suggests that the TFs identified by TIDAL are likely to have broad importance. In addition to confirming the involvement of known factors, TIDAL predicts several new antiviral TFs and regulatory connections that are candidates for experimental follow-up. CREB1 is identified as a component of the measles response (by both TIDAL and DREM). The CREB transcription factor has been proposed as an inhibitor of NF-kB activity [41]. A second intriguing case in the measles network is ZEB1, which was also previously predicted to participate in the NDV (Newcastle Disease Virus) response [26]. ZEB1 has been shown to suppress IL-2, a key immune cytokine [42]. In previous work [26], we have used electrophoretic mobility shift assays (EMSA) to follow up on similar computational predictions, and validated several novel antiviral regulatory connections.

Visualization of the dynamic transcriptional networks produced by TIDAL presents several challenges. Figures 1 and 3 present two complementary views of the regulatory network, and both are required to gain an accurate picture of the response. Figure 1 shows the temporal activity profile associated with each transcription factor binding site matrix. From this visualization, it is clear that a significant amount of transcriptional activity is concentrated early in the response. However, Figure 3 shows that the up-regulation of TF genes associated with this activity is spread throughout the response. It is possible that these TF are indeed up-regulated earlier, but that our criteria for differential expression are too conservative and they are assigned to later time-points. Indeed, it is known that TFs are often active at lower expression levels than other genes [43]. Another possibility is that these TFs are initially activated through post-translational mechanisms, and only later become up-regulated at the transcriptional level. Future work should explore these possibilities along with better ways to integrate this information visually into a single view.

In summary, we have developed TIDAL, a new method for constructing transcriptional regulatory networks from time-series transcriptional profiling data. Application of our integrative analysis to data from the influenza and measles responses enables us to identify the underlying regulatory network structure, along with potentially novel antiviral transcription factors. Importantly, TIDAL provides specific hypotheses that can be validated experimentally. These hypotheses take the form of: Transcription factor *A *regulates target gene *B *at time *T *through the binding site *S *located at position *P *in the promoter region. Moreover, our time-centric approach is generally applicable to understanding the immune response to other pathogens. Such reconstructions of transcriptional networks underlying the immune response across infections and cell types, coupled with the ability to compare them, will provide critical insight into the host immune response and viral antagonism.

## Methods

### Differentiation of DCs

All human research protocols for this work have been reviewed and approved by the IRB of the Mount Sinai School of Medicine. Monocyte-derived DCs were obtained from healthy human blood donors following a standard protocol described elsewhere [39]. Briefly, human peripheral blood mononuclear cells were isolated from buffy coats by Ficoll density gradient centrifugation (Histopaque, Sigma Aldrich, St. Louis, MO) at 1450 r.p.m. and CD14+ monocytes were immunomagnetically purified by using a MACS CD14 isolation kit (Miltenyi Biotech, Bergisch Gladbach Germany). Monocytes were then differentiated into naïve DCs by 5 day incubation at 37 °C and 5% CO2 in DC growth media, which contains RPMI Medium 1640 (Invitrogen/Gibco, Carlsbad CA) supplemented with 10% fetal calf serum (Hyclone, Logan UT), 2 mM of l-glutamine, 100 U/ml penicillin and 100 g/ml streptomycin (Pen/Strep) (Invitrogen, Carlsbad CA), 500 U/ml hGM-CSF (Preprotech, Rocky Hill NJ) and 1000 U/ml hIL-4 (Preprotech, Rocky Hill NJ).

### Virus preparation and viral infection

The recent seasonal H1N1 influenza virus A/New Caledonia/20/1999 (NC) was kindly provided by Dr. Peter Palese and grown in 10- to 11-day-old embryonated chickens eggs as described previously [44]. The Virus was titrated by immunofluorescence 18 hours after infection of MDCK cell plates using monoclonal antibodies specific for Influenza-NP protein generated by the Mount Sinai Hybridoma Core Facility followed by addition of anti-mouse IgG-FITC and visualization using fluorescent microscopy. For infection of naïve DCs, NC stocks were appropriately diluted in Dulbecco's Modified Eagle Medium (DMEM) and added directly into pelleted DCs at a multiplicity of infection (MOI) of 1 [39,45]. After incubation for 40 minutes at 37°C, fresh DC growth medium (without GMCSF and IL-4) was added back to the infected cells (1 × 106 cells/ml) for the remainder of the infection. The reaction was stopped at 1, 2, 4, 6, 8, 10, and 12 hours after infection by fixing the cells with RNAprotect Cell Reagent (Qiagen, Duesseldorf Germany). Naïve non-infected DCs underwent the same experimental procedure as infected DCs in the absence of virus to ensure that mechanical manipulations could not be responsible for differences in experimental readouts. All time points and controls were performed in triplicates.

### RNA extraction

Cells were homogenized by using QIAshredder microcentrifuge spin-columns (Qiagen, Duesseldorf Germany) and RNA was isolated from cells using Qiagen Micro RNeasy plus kit following the manufactures protocol (Qiagen, Duesseldorf Germany). RNA quality was assayed by determination of the RNA integrity number using the 2100 Bioanalyzer (Agilent).

### Microarray analysis

RNA samples were processed and hybridized to HumanHT-12 v4 Expression BeadChip Kit (Illumina San Diego, CA) by the Mount Sinai Genomics Institute following the manufacturer's instructions, and raw expression data were output by the Illumina GenomeStudio software. Microarray data are available through the Gene Expression Omnibus (GEO) Database, accession number GSE41067. The data were log-transformed and quantile normalized [46]. Differential expression was defined for each probe at each infection time-point using three criteria: (1) a minimum intensity of 128, (2) an absolute fold-change of at least two relative to control, (3) a significant change in expression by LIMMA (BioConductor [47] implementation) after correction for multiple hypothesis testing (*q <*0.05). In analysis where a more inclusive gene universe is used, it was defined as the set of genes from all time-points that met criteria 1 and 3 (minimum observed intensity and significant change by LIMMA). This definition provided an expanded set to allow for more power in statistical enrichment tests, but ensured that all genes exhibited some changes over the response. All of this analysis was performed using BioConductor software packages [47] in R. Few genes were found to be up-regulated at the 1 hour time-point post-infection, and it was omitted from further analysis.

### Transcription factor target identification

Using the UCSC Genome Bioinformatics site, we downloaded the transcription start site data (TSS) for all human RefSeq genes, defined by the January 2010 refGene table [48]. The region +/-2Kb around each TSS was identified within a genome-wide multiple alignment of 45 vertebrate species to the human genome [49], also available through the UCSC Genome Bioinformatics site. In order to identify putative transcription factor binding sites, the human sequences, along with aligned regions from mouse, were masked for repetitive elements using RepeatMasker [50] and then analyzed using the TRANSFAC MATCH [51] algorithm with a cutoff, as defined within the database, chosen to minimize the sum of false positives and false negatives. The analysis was performed for all high quality vertebrate transcription factor matrices in the 2011.1 release of TRANSFAC [28], and putative binding sites were considered to be evolutionarily conserved if matches were also found at the aligned positions in the mouse sequences and had no gaps present in the multiple alignment between the species being compared. Each TRANSFAC matrix was linked to a set of gene symbols describing potential binding factors using annotations present in the "Binding Factor" field of the database. Only vertebrate TRANSFAC matrices that could be linked to a HGNC gene symbol, either directly or through an alias listed in NCBI gene, were included.

### Inferring transcriptional regulators of each time slice

The TF inference was based on statistical enrichment of putative TF targets. More precisely, we defined *G*, called the foreground set, to be the set of genes first up-regulated at a particular time-point, and *T *to be the set of all genes in the dataset with binding sites for a given TRANSFAC matrix *M*. The set *T *is determined by choosing an appropriate sequence match cutoff for matrix *M*, and further filtering the genes with matches to M, we included only those with a conserved binding site for an orthologous mouse and chimp gene (as described above). The background set *B*, which served to catalog the expected distribution of binding sites for *M *and dictate whether the observations in the foreground set *G *are unusual, is computed as the set difference between the gene universe *U *(see Microarray analysis above) and the foreground set *G *under consideration. Note that the background set changed slightly depending upon the time slice under analysis. Next we found how many of the genes in the foreground and the background contained binding sites for the matrix *M*. That defined subsets in the foreground and the background as intersections of *G *and *B*, respectively, with *T*. Defining $N_{g}=\left|G\right|$, $N_{b}=\left|B\right|$, $H_{g}=\left|G\cap T\right|$ and $H_{b}=\left|B\cap T\right|$, we computed the hypergeometric P-value, which is the probability that, if binding sites for M were assigned randomly to genes in the foreground and the background, one might observe at least *H_g _*binding sites in the foreground set. We computed such P-values for every TRANSFAC matrix mapped to a gene found to be up-regulated somewhere in the time-course at each time-point, and retained those passing an FDR-corrected threshold of 0.05.

### Important network connections

To connect the individual TFs into a regulatory network, we placed each gene represented by one of the enriched matrices at the time it is first determined to be differentially expressed. For every TF included in the network, we defined an activity window for it. Contained in this window was the consecutive stretch during which the gene is up-regulated in the microarray, possibly extended to include all time-points of enrichment. Once the activity window had been identified, the transcription factor was connected explicitly to all nodes in the network with binding sites for the factor and implicitly with all other non-TF targets, placed within its activity window. Connections from a node placed earlier in time to nodes in later time-points were referred to as forward links; the reverse is true for back links.

Finally, we selected the more important links among all the network connections. Suppose the TF in question was placed in the network at time *t*. To choose the most likely regulators, for each TF *R_i _*we considered the time interval during which *R_i _*is significantly enriched (as shown in Figure 1), and selected the time-point *t_i _<*= *t *that was closest to *t*. Using the time *t_i _*assigned to TF *R_i_*, we chose the TFs closest to *t *as the regulators. Enrichment P-values were used for breaking ties among TFs with the same chosen time assignment. On occasion, when this scheme resulted in selecting more regulators than specified by parameters (such as the four regulators for the node Irf1 in Figure 3), all such regulator links were kept without making arbitrary omissions. Note that no limits were placed on the number of outgoing links for each node.

## Additional file

Additional file 1 provides details of our analysis of the DC response to measles infection using TIDAL.

## List of abbreviations

DCs: monocyte-derived dendritic cells; TF: transcription factor; TIDAL: *TI*me-*D*ependent *A*ctivity *L*inker; DREM: Dynamic Regulatory Events Miner; NDV: Newcastle Disease Virus

## Competing interests

The authors declare that they have no competing interests.

## Authors' contributions

EZ designed and implemented the network reconstruction method, and analyzed the data. GN performed the DREM analysis. UH helped design the methods. SM carried out the analysis of binding site locations, BMH performed the experiments, JT carried out the influenza microarray analysis and SHK carried out the measles microarray analysis. SCS and SHK conceived the study. SHK designed the methods. EZ and SHK wrote the manuscript.

## Supplementary Material

Additional file 1 Click here for file

## Declarations

This article has been published as part of *BMC Bioinformatics *Volume 14 Supplement 6, 2013: Selected articles from the 10th International Conference on Artificial Immune Systems (ICARIS). The full contents of the supplement are available online at http://www.biomedcentral.com/bmcbioinformatics/supplements/14/S6.
