# Supplementary material for: Reconstruction of regulatory networks through temporal enrichment profiling and its application to H1N1 influenza viral infection
Source: BMC Bioinformatics. 2013 Apr 17;14(Suppl 6):S1. doi: 10.1186/1471-2105-14-S6-S1 (PMC3633009; doi:10.1186/1471-2105-14-S6-S1)
Supplement: Additional file 1 [file 1471-2105-14-S6-S1-S1.pdf]

In this article we describe TIDAL, a computational methodology for inferring transcriptional regulatory cascades in infection time-courses. The main body of the manuscript presents our analysis of the influenza response. Here we provide details of our analysis of the DC response to measles infection [1]. Raw Affymetrix microarray data were downloaded from GEO (GSE980). Data normalization and differential expression procedures follow the same pipeline as stated in *Methods*. However, to account for differences in the microarray hybridization profiles, we use a minimum expression intensity of 32 for the measles analysis. In addition, a false discovery rate cutoff of $q < 0.1$ is used to be consistent with the original study [1]. The measles time-course includes a pre-infection (control) time point, along with samples at 3, 6, 12 and 24 hours post-infection. We analyse the measles dataset using the 2008.3 release of TRANSFAC [2]. Putative binding sites are filtered for evolutionary conservation with chimp and mouse sequences.

Identification of factors driving the measles response. Using our differential expression analysis, we identify a total of 937 genes up-regulated over the course of the measles response (Supplementary Figure 1). Temporal activity profiling finds 42 TRANSFAC matrices with significant activity (Supplementary Figure 1). Similar to the influenza response, these inferred matrices are associated with many known integral components of the antiviral response, including those linked with interferon activation and signaling (IRFs, STATs, NFkB) [3]. Several novel factors are also identified.

Binding site location analysis. We find a more significant shift in binding site locations at times of peak predicted activity (e.g., 3 hours post-infection for the IRF matrix) compared with times of minimum activity. Supplementary Figure 2 shows this behavior for the specific case of IRF, as well as over the set of all binding matrices with inferred activity. Inspection of the location plots shows that the shift in location during times of TF activity is towards the TSS, as expected. This behavior is consistent with our observations for the influenza response (Figure 2 in the main text), once again suggesting that true transcription factor targets are likely to be found during times of significant target enrichment.

Measles antiviral response network. After filtering the set of potential regulator-target links using TF activity windows and limiting the incoming links of each node to its three most likely regulators, we obtain a network that contains 34 TF nodes and 96 regulatory links (Supplementary Figure 3). As with the influenza response network, a majority of the inferred links in the network are forward links (colored green). All but two of the TFs could be connected together into a single network. The two remaining TFs, which have inferred activity, but are disconnected from the rest of the network by our regulatory link filtering procedure, are removed. The network accounts for 82% of all the genes up-regulated during the measles response.

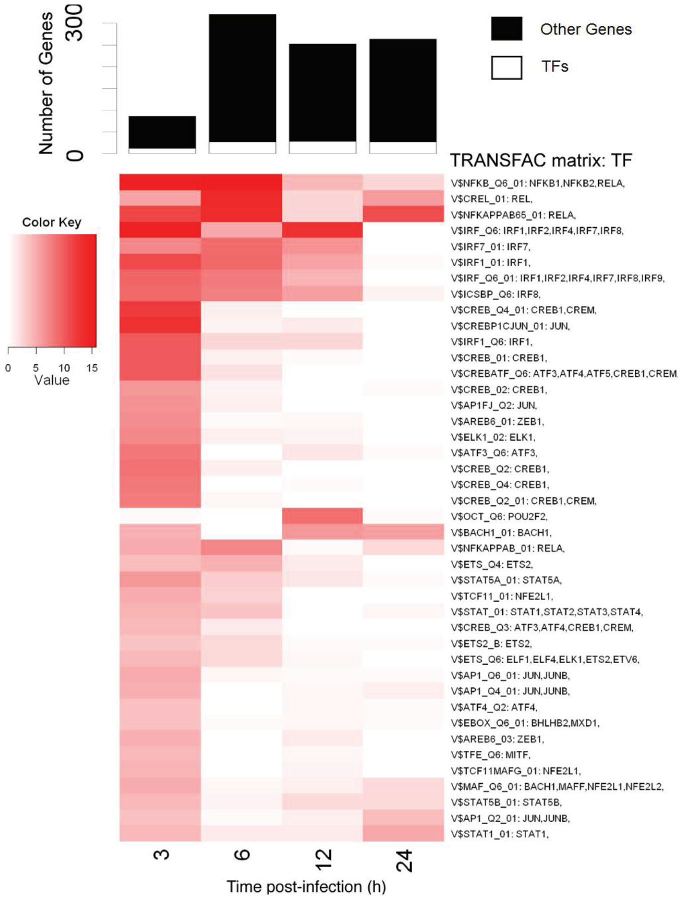

Supplementary Figure 1: **Enrichment profiles for TFs implicated in the measles response.** Top panel shows numbers of genes first up-regulated at each time point, split by TF and non-TF genes. Bottom panel shows a heatmap plot with the over-representation analysis of targets associated with each of the TRANSFAC matrices (rows) over time (columns). Only matrices with inferred activity are pictured, and the color ($-log$(`p-values`)) indicates the significance of the hypergeometric test. The associations between a TRANSFAC matrix and the individual (up-regulated) TFs are shown to the right of the color profile.

Comparison with DREM. We compare the performances of TIDAL and DREM [4] on the measles gene expression data. Using the same set of TF matrices and their mapped binding sites as in TIDAL, DREM identifies 6 distinct temporal profiles (see Supplementary Figure 4). However, regulators are identified for only 3 of the 9 clusters leading out of branching points. Virtually all of the regulators identified by DREM are also found by TIDAL (Supplementary Figure 1). To compare the precision and recall of the methods, we compute the overlap between the TFs inferred by TIDAL (pictured in Supplementary Figure 3) and DREM (mapped from the TRANSFAC matrices pictured in Supplementary Figure 4) with a set of known immune response genes [5, 6]. Precision for the two methods is almost identical: 67% (10/15) of the TF

genes annotated to matrices identified by DREM are associated with known TFs, and 66% (23/35) of TF genes identified by TIDAL are known. However, recall for TIDAL stands at 30%, as compared with 13% for DREM. Thus, as with the influenza response (Figure 5 in the main text), DREM and TIDAL exhibit similar precision, but TIDAL has higher recall.

Overall, DREM identifies the main drivers of the measles response. It associates the NFkB and IRF family TFs with a group of highly up-regulated genes in the early phase of the response. DREM also finds three TRANSFAC matrices with activity at 12 hours post-infection. One of these, V$TCF11MAFG_01 that is associated with the NFE2L1 gene, is also identified as having modest activity at this time by TIDAL. However, TIDAL finds that the activity of V$TCF11MAFG_01 at 3 hours is even more pronounced. Interestingly, DREM associates this activity with a decrease in expression of previously up-regulated genes, while TIDAL predicts involvement with up-regulation. It is possible that this TF has differential effects on different genes and that the two methods are picking up complementary signals. TIDAL also implicates several related matrices (V$TCF110_1 and V$MAFF_Q6_01) showing activity patterns analogous to that of V$TCF11MAFG_01. Similarly, at 24 hours post-infection, DREM implicates V$AP1_Q4_01, while TIDAL infers modest activity for the matrix at that time, with the main activity happening earlier in the response. Again, several related matrices are picked up by TIDAL as well. While DREM identifies two of the most well-known TF families in the antiviral response (namely IRF and NFkB), the STATs are conspicuously absent. In contrast, STAT family activity is identified by TIDAL during the first 6 hours post-infection, consistent with known biology [3].

References

1. Zilliox M, Parmigiani G, Griffin D: **Gene expression patterns in dendritic cells infected with measles virus compared with other pathogens**. *PNAS* 2006, **103**(9):3363–3368.

2. Matys V, Fricke E, Geffers R, Gössling E, Haubrock M, Hehl R, Hornischer K, Karas D, Kel AE, Kel-Margoulis OV, Kloos DU, Land S, Lewicki-Potapov B, Michael H, Münch R, Reuter I, Rotert S, Saxel H, Scheer M, Thiele S, Wingender E: **TRANSFAC: transcriptional regulation, from patterns to profiles**. *Nucleic Acids Res* 2003, **31**:374–378.

3. Haller O, Kochs G, Weber F: **The interferon response circuit: Induction and suppression by pathogenic viruses**. *Virology* 2006, **344**:119–130.

4. Ernst J, Vainas O, Harbison C, Simon I, Bar-Joseph Z: **Reconstructing dynamic regulatory maps**. *Nature-EMBO Molecular Systems Biology* 2007, **3**:74.

5. Huang Q, Liu D, Majewski P, Schulte L, Korn J, Young R, Lander E, Hacohen N: **The plasticity of dendritic cell responses to pathogens and their components**. *Science* 2001, **294**(5543):870–875.

6. Jenner R, Young R: **Insights into host responses against pathogens from transcriptional profiling**. *Nat Rev Microbiol.* 2005, **3**(4):281–294.

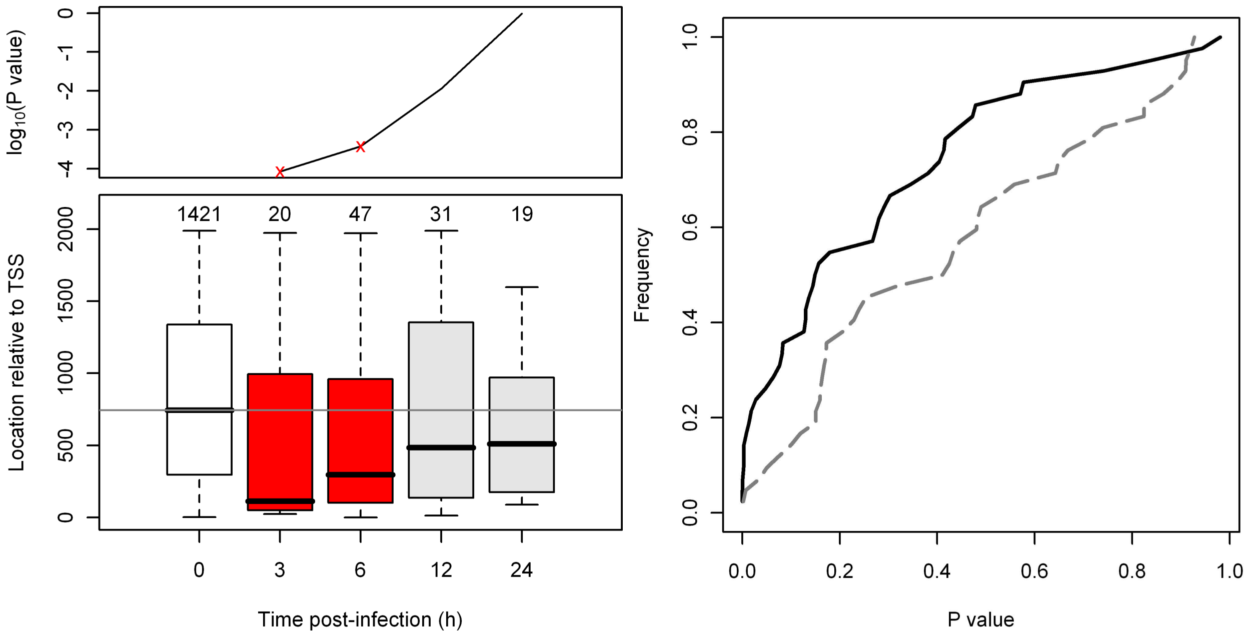

(a) (b)

Supplementary Figure 2: **Binding site location mirrors TF activity profile.** (a) Analysis of the general IRF matrix (IFR_Q6_01). Top panel shows profile for the over-respresentation analysis, with red `x` indicating significance at 3 and 6 hours post infection (FDR $q < 0.05$). Bottom panel shows Boxplots of the absolute locations of binding sites in promoter regions of genes first up-regulated at each time point. The number of genes in each group is indicated above the box. The medians are indicated by horizontal bars. The box at time zero shows a control group of genes, and contains all RefSeq genes with binding sites for the same matrix that are not differentially-expressed. Red bars indicate that the median binding site position is significantly closer to the TSS as compared with the control group ($P < 0.05$, Mann-Whitney test). When multiple binding sites for a matrix are present for a single gene, the one closest to the TSS is selected. (b) Plots the cumulative distributions of the P-values, testing for differences in binding site locations as described in (a), at the time of minimum enrichment P-value (highest inferred TF activity, solid line) and maximum enrichment P-value (least inferred TF activity, dashed line). We observe a significant shift in the locations distribution ($P < 0.01$, Mann-Whitney test) toward the TSS during times of TF activity.

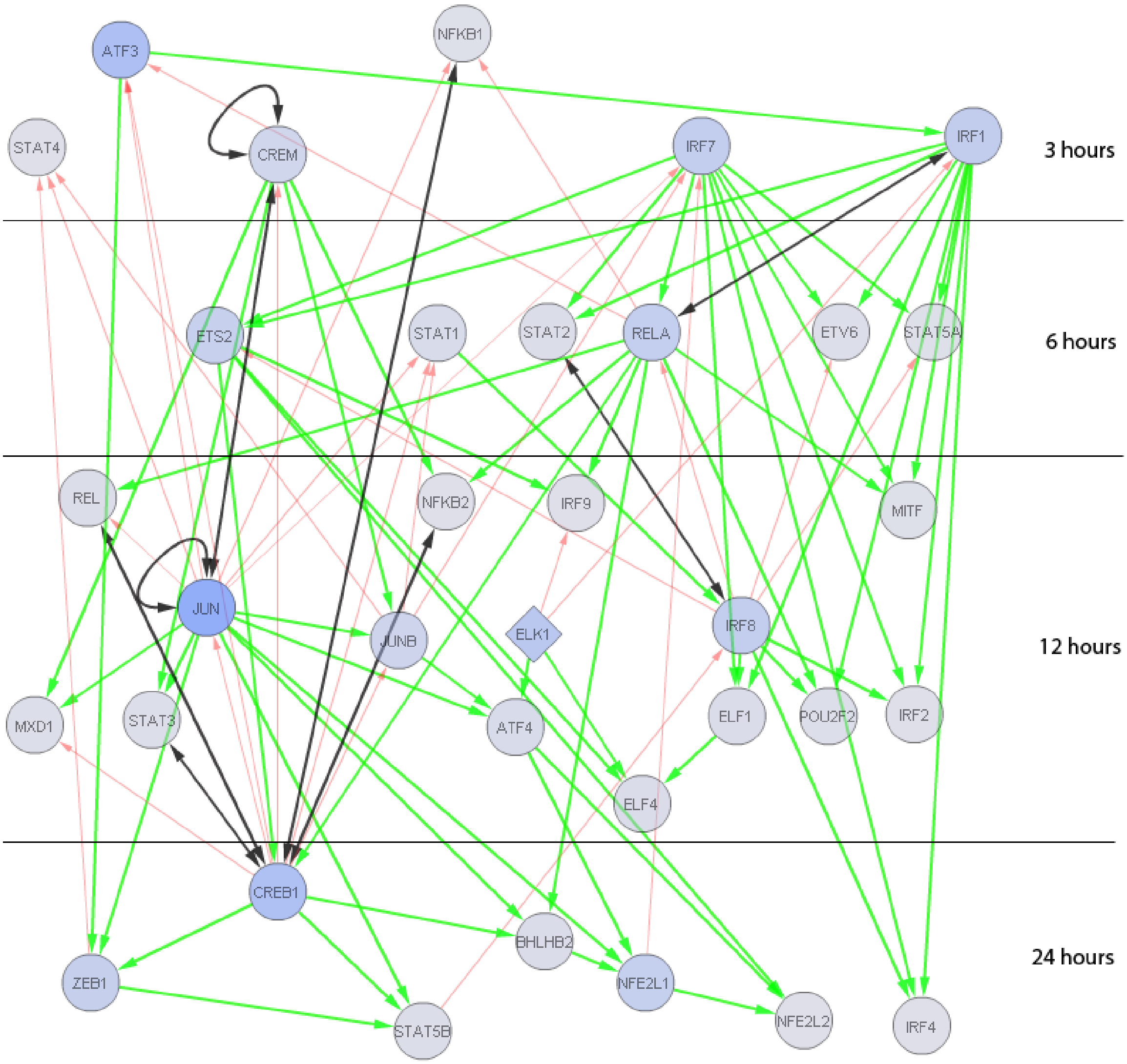

Supplementary Figure 3: **The measles regulatory response network.** Network nodes correspond to individual TFs. Edges indicate predicted regulatory relationships, which can be either forward (green links), feed-back (red links) or reciprocal (black links). Time in the figure progresses vertically down, with nodes placed in the time-slice during which the gene is first differentially expressed. Nodes that were not observed to be transcriptionally regulated by other network members are diamond shaped. The network edges are filtered to include at most three predicted regulators for each target.

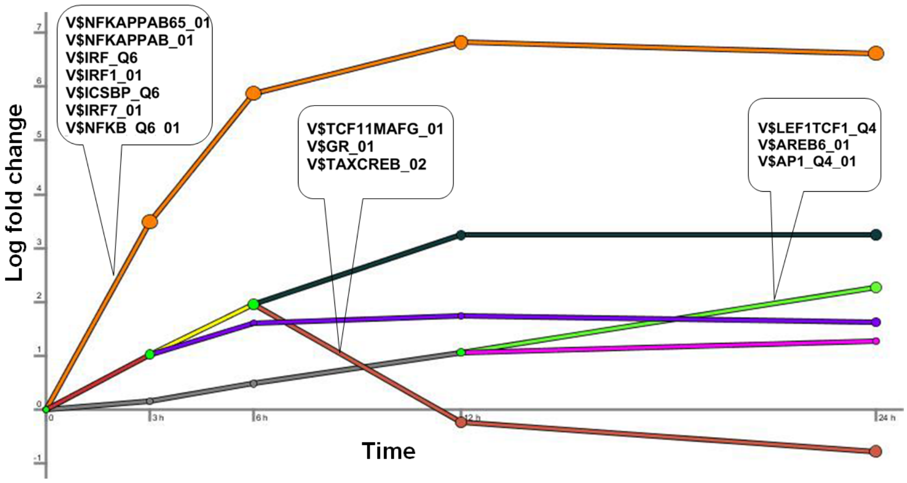

Supplementary Figure 4: **Dynamic regulatory map of the measles response.** DREM [4] was used to create a dynamic map based on the time-series fold-change gene expression data as described in Methods. DREM parameters were left at their defaults. Each line in the figure represents a temporal cluster. Predicted regulatory events (cut-off of 10^{-3}) are indicated pointing to the temporal profile immediately after the split they regulate.
